# A scoring system to effectively evaluate central nervous system tuberculosis in patients with miliary tuberculosis

**DOI:** 10.1371/journal.pone.0176651

**Published:** 2017-05-22

**Authors:** Yongjiu Xiao, Shuqing Yu, Qingliang Xue, Shan Lang, Junping Sun, Dan Feng, Jianxin Wang

**Affiliations:** 1 Department of Respiratory Disease, Chinese PLA General Hospital, Beijing, China; 2 Department of Respiratory Disease, Lanzhou General Hospital of Chinese PLA, Lanzhou, Gansu Province, China; 3 The Second Ward of Lanzhou Pulmonary Hospital, Lanzhou, Gansu Province, China; Temple University School of Medicine, UNITED STATES

## Abstract

There is currently no convenient way to effectively evaluate whether a miliary tuberculosis patient is complicated with central nervous system (CNS) tuberculosis. We aimed to find such a way by analyzing the clinical data of these patients. Fifty patients with confirmed miliary tuberculosis and 31 patients with confirmed miliary tuberculosis complicated with CNS tuberculosis from 2010 to 2014 were selected. Their general conditions, clinical features and laboratory tests were analyzed. Factors that were significantly different between them were chosen to performed multivariate and univariate logistic regression analyses, and factors with significant P values were used to establish a scoring system. Eight factors, i.e., age, cough, nausea, headache, hemoglobin (HGB), serum albumin (ALB), C-reactive protein (CRP) and erythrocyte sedimentation rate (ESR), were significantly different (*P* < 0.05). Multivariate logistic regression analysis showed that ALB was the independent risk predictor (HR = 1.29, 95% CI 1.09–1.52, *P* < 0.01), whereas the others were non-independent predictors except age (*P* < 0.05). The scoring system was based on a summation of the scores of the assigned values of the seven predictors and had an area under the curve (AUC) of 0.86 to confirm CNS tuberculosis, with a sensitivity of 81.5% and a specificity of 81.4% at a score of 0.75 and with a specificity of 95.3% at a score of 2.75. In contrast, a score below -0.75 excluded CNS tuberculosis, with a sensitivity of 88.9% and a specificity of 62.7%. The scoring system should be useful to evaluate whether a miliary tuberculosis patient is complicated with CNS tuberculosis and could help doctors avoid excessive investigation.

## Introduction

Tuberculosis (TB) caused by the bacillus *Mycobacterium tuberculosis* is prevalent all over the world and is characterized as the deadliest communicable disease [1]. In the 2015 World Health Organization (WHO) tuberculosis report, the number of global cases reached 9.6 million, and 1.5 million people were killed; TB had a mortality rank of 5, following ischemic heart disease, stroke, lower respiratory infections and chronic obstructive disease. Even worse, approximately 70% of all smear-positive pulmonary TB patients without anti-TB treatment died within 10 years, and more than 30% of the patients with HIV-associated TB died in 2014 [1–4]. Hence, it is also considered one of the most important threats in the world, and a cure to this curable disease is far from existing [5]. In the meantime, however, early diagnosis and timely treatment are critical for patients’ progression because delay in treatment is strongly associated with death. Thus, the British infection society guidelines suggested beginning treatment once TB is diagnosed clinically, without the microbiological or molecular diagnostic confirmation [6, 7].

Pulmonary TB (PTB), as the most important infectious source of intra/extrapulmonary TB, is a main component of TB because of its proportion of more than 80% in all new TB patients or in TB patients whose previous treatment status is unknown [1]. The routine diagnosis of PTB is based on clinical signs and symptoms, chest X-ray/computed tomography (CT), Mantoux tuberculin skin test, and the definitive evidence of *M*. *tuberculosis* in clinical samples or cultures, including sputum, bronchoalveolar lavage fluid, and bronchoscopic or tissue biopsy [8–10]. Other methods such as interferon-γ release assays and blood tests to detect antibodies alone are not recommended in active TB but are recommended in TB-latent people with a positive Mantoux test [11–13].

Miliary tuberculosis and miliary tuberculosis complicated with CNS tuberculosis develop from the intra/extrapulmonary tubercle bacilli that spread throughout the lungs or to the central nervous system via the blood in the vascular system [14, 15]. In addition to the diagnostic steps of PTB, the characteristic imaging of diffuse and tiny lesions in the lung, which are directly visible on chest X-ray or CT imaging, is the basic diagnostic evidence for miliary tuberculosis [1, 8, 16, 17]. CNS tuberculosis is a normal neurological involvement that includes tuberculous meningitis, tuberculoma, tuberculous abscess, and nonosseous spinal tuberculoma and occurs at a very high proportion (38.5%) in patients with miliary tuberculosis. It is more dangerous in miliary tuberculosis patients because the mortality rate is over 30% and has reached 41% in previous reports [16–19]. Thus, aggressive and timely treatment becomes necessary because it is very important for the prognosis. Head CT/magnetic resonance imaging (MRI) scan or cerebrospinal fluid analyses are routinely performed to avoid missing a diagnosis or providing a misdiagnosis in patients with miliary tuberculosis [20]. However, radiographic examination and collecting cerebrospinal fluid via lumbar puncture increase the costs and delay the time of diagnosis and treatment, and lumbar puncture even has the potential to injure the spinal nerve or cause infection. How, thus, can doctors objectively evaluate miliary tuberculosis patients complicated with CNS tuberculosis without these examinations and avoid excessive investigation? Many studies have shown decreased levels of HGB and ALB, elevated levels of adenosine deaminase (ADA) and CRP, and increased ESR in miliary tuberculosis patients. These factors are considered predictors for miliary tuberculosis but are rarely mentioned in miliary tuberculosis patients with CNS tuberculosis [21–28]. We postulated that these differences might be predictors of CNS tuberculosis in patients with miliary tuberculosis. Hence, we collected and analyzed the general conditions, clinical features and laboratory tests of patients with confirmed miliary tuberculosis and those complicated with CNS tuberculosis to achieve this goal.

## Methods

### Patients

This was a retrospective, observational cohort study. The data were collected from Lanzhou Pulmonary Hospital, a hospital that was designated by the government for diagnosing and treating tuberculosis. The ethics committee of the Pulmonary Hospital approved this study (Approval Number: LZFK150812). The study does not need the informed consent from participants because it is a retrospective study from case history. The study does not involve tissue samples or other fluid specimens from the participants. All authors had access to information that could identify individual participants after data collection. In our retrospective study, patients diagnosed with miliary tuberculosis first were screened from the case history database. Patients complicated with CNS tuberculosis, including intracranial and spinal tuberculosis, were selected from the initially screened patients. Overall, 50 patients with miliary tuberculosis (group A) and 31 patients with miliary tuberculosis and CNS tuberculosis (group B) from January 2010 to January 2014 were confirmed who fit the inclusion criteria and had used the same equipment for laboratory tests. All these screened patients were HIV negative.

### Diagnostic and differential diagnosis criteria

Briefly, the diagnosis of miliary tuberculosis met the following criteria: (1) possible clinical symptoms of one or several of the following such as cough, sputum, fever, night sweats, fatigue, emaciation, nausea, vomiting, chest pain, and shortness of breath; (2) images showing diffuse, bilateral and small lesions (from 1 to 3 mm in diameter) in the lung by chest X-ray or HRCT scan [29–31]; (3) positive microscopic samples for acid-fast bacilli using the Ziehl-Neelsen stain in sputum, lung alveolar fluids, tracheal and lung biopsy tissues, or positive cultures of the above samples [32, 33]; (4) negative examination of the cerebrospinal fluid; (5) each laboratory test detected using the same equipment; and (6) a poor positive rate of *mycobacterium tuberculosis*, and the diagnosis of most patients depended on the coordination of the imaging and clinical features. The diagnosis of CNS tuberculosis in patients with miliary tuberculosis included all of the above criteria except (4) with additional criteria: (1) positive images of the spine or head by CT/MRI scan and (2) positive examination of the cerebrospinal fluid, including moderate lymphocytic pleocytosis, moderately elevated protein levels, and hypoglycorrhachia (low glucose) and, in particular, positive results for acid-fast bacilli [34, 35]. The above diagnoses and the differential diagnoses without definitive evidence of *Mycobacterium tuberculosis* were confirmed by two independent medical specialists. The exclusion criteria were any symptoms of cough, sputum, fever, night sweats, fatigue, emaciation, nausea, vomiting, chest pain, shortness of breath or headache caused by other diseases.

### Clinical and laboratory procedures

Data concerning the clinical procedures such as general conditions, clinical features, and laboratory tests were collected. General conditions included gender, age and duration of illness. Clinical features included cough, sputum, fever, night sweats, fatigue, emaciation, nausea, vomiting, chest pain, shortness of breath and headache. Laboratory tests included routine blood tests, biochemical assays and inflammatory factor tests. Routine blood tests included the white blood cell count, red blood cell and platelet counts, neutrophil percentage, and HGB level. Biochemical assays included the levels of alanine aminotransferase (ALT), aminotransferase (AST), γ-glutamyl transferase (GGT), serum total protein (TP), ALB, serum globulin (GLB), total bilirubin (TBIL), direct bilirubin (DIBL), indirect bilirubin (IBIL), urea nitrogen (BUN) and creatinine (Cr). Inflammatory factor tests included the levels of ADA, ESR, and CRP.

### Statistical analysis

All data were input and analyzed using Statistical Product and Service Solutions (version 13.0, Chicago, Illinois, USA). Student’s t-test was used for continuous data (e.g., age) to determine whether the median levels were different in the two groups. The chi-square test was used for categorical data such as gender and clinical symptoms. Fischer’s exact probability was calculated if there were fewer than five cases. Multivariate and univariate logistic regression were used to screen the influential factors, and the receiver operating characteristic curve (ROC) was used to identify the sensitivity and specificity. p-values were two-sided with α< 0.05.

## Results

### Subject characteristics

In total, 138 cases of confirmed miliary tuberculosis were screened, but only 81 fit the inclusion criteria. **Table 1** shows that there were 29 (58%) male patients and 21 (42%) female patients in group A, and 13 (42%) male patients and 18 (58%) female patients were in group B. No significant difference in gender was found between the groups (*P* = 0.160). There also was no significant difference in the median duration of illness between the groups (47.58±47.03 *vs*. 50.32±40.75, *P* = 0.789), but there was a significant difference in the median age (30.50±20.00 *vs*. 23.74±9.87 years, respectively, *P* = 0.046).

**Table 1:** Comparison of general conditions and clinical manifestations between the groups.

	Group A (n = 50)	Group B (n = 31)	*P*
**General conditions**			
male	29(58%)	13(42%)	0.160
female	21(42%)	18(58%)	
duration of illness	47.58±47.03	50.32±40.75	0.789
age	30.50±20.00	23.74±9.87	0.046
**Clinical manifestations**			
cough	33(66%)	11(35%)	0.011
sputum	17(34%)	6(19%)	0.207
fever	45(90%)	24(77%)	0.197
night sweats	8(16%)	4(13%)	0.760
fatigue	10(20%)	7(23%)	0.786
emaciation	3(6%)	1(3%)	1.000
nausea	5(10%)	12(39%)	0.004
vomiting	5(10%)	8(26%)	0.070
chest pain	3(6%)	0	0.282
shortness of breath	11(22%)	4(13%)	0.386
headache	11(22%)	21(68%)	0.000

Data are median (IQR), number (%). (Measurement data for t-test, categorical data for chi-square test, number<5 for Fischer’s exact probability).

Clinical features, including cough, sputum, fever, night sweats, fatigue, emaciation, nausea, vomiting, chest pain, shortness of breath and headache, are compared in **Table 1**. Fever was the most common clinical feature, with a proportion of 90% in group A and 77% in group B, but there was no significant difference between the groups (*P* = 0.197). Cough was a common symptom in group A, and its frequency was significantly higher than that in group B (66% *vs*. 35%, *P* = 0.011). On the contrary, the frequencies of headache and nausea were much higher in group B than in group A (68% *vs*. 22%, 39% *vs*. 10%, respectively, *P* < 0.01). No significant differences were found in other symptoms.

### Laboratory tests

Routine blood tests, biochemical assays and inflammatory factor examinations are compared in **Table 2**. Group A had a morbidity of anemia of more than 50%, which was much higher than that in group B (less than 30%, *P* < 0.05), and the median level of HGB was significantly lower than in group B (*P* < 0.05). Biochemical assays showed that the median levels of ALT and AST in both groups were higher than the upper limit of the normal range but with no significant differences between them (*P* > 0.05) or in the median levels of TBIL, DBIL, and IBIL (*P* > 0.05). The median levels of ALB and TP in group A were significantly lower than those in group B (31.70±.68 *vs*. 40.03±6.38, 60.87±12.21 *vs*. 68.42±11.79, respectively, *P* < 0.05), but the median level of GLB was not significantly different (29.17±10.54 *vs*. 28.40±11.18, *P* = 0.775). The levels of Cr and BUN in all patients were in the normal range and showed no significant differences between the two groups. However, the median levels of CRP and ESR in group A were much higher than those in group B (*P* < 0.05), but no significant difference was found in the median level of ADA (*P* = 0.134).

**Table 2:** Comparison of routine blood tests, biochemical assays and inflammatory factors between the groups.

	Group A (n = 50)	Group B (n = 31)	*P*
Blood routine test			
White blood cells (×10^9^/L)	6.81±2.72	6.60±2.80	0.737
Neutrophils %	74.27±12.31	71.76±12.29	0.377
Red blood cells (×10^12^/L)	4.24±0.75	4.48±0.58	0.131
Hemoglobin (g/L)	115.61±20.78	126.87±18.13	0.015
Platelets (×10^9^/L)	244.47±104.13	223.68±89.96	0.363
Biochemical assay			
Alanine aminotransferase (U/L)	40.18±33.81	51.26±65.05	0.321
Aminotransferase (U/L)	48.96±74.68	48.10±79.70	0.961
γ-glutamyl transferase (U/L)	69.49±69.25	86.04±88.71	0.392
Serum total protein (g/L)	60.87±12.21	68.42±11.79	0.015
Serum albumin (g/L)	31.70±6.68	40.03±6.38	0.000
Serum globulin (g/L)	29.17±10.54	28.40±11.18	0.775
Total bilirubin (μmol/L)	18.89±14.91	16.44±8.13	0.405
Direct bilirubin (μmol/L)	8.25±6.02	6.78±4.43	0.244
Indirect bilirubin (μmol/L)	10.15±8.12	9.66±4.82	0.759
Urea nitrogen (mmol/L)	4.00 ±1.36	3.48±1.41	0.142
Creatinine (μmol/L)	55.15±41.25	41.79±9.50	0.123
Inflammatory factors			
Adenosine deaminase (U/L)	29.04±36.45	17.57±7.93	0.134
Erythrocyte sedimentation rate (mm/h)	49.78±32.33	27.92±24.31	0.005
C-reactive protein (mg/L)	29.01±51.17	7.25±12.66	0.013

Data are median (IQR), all the measurement data for independent t-test.

### Scoring system establishment

**Fig 1:**
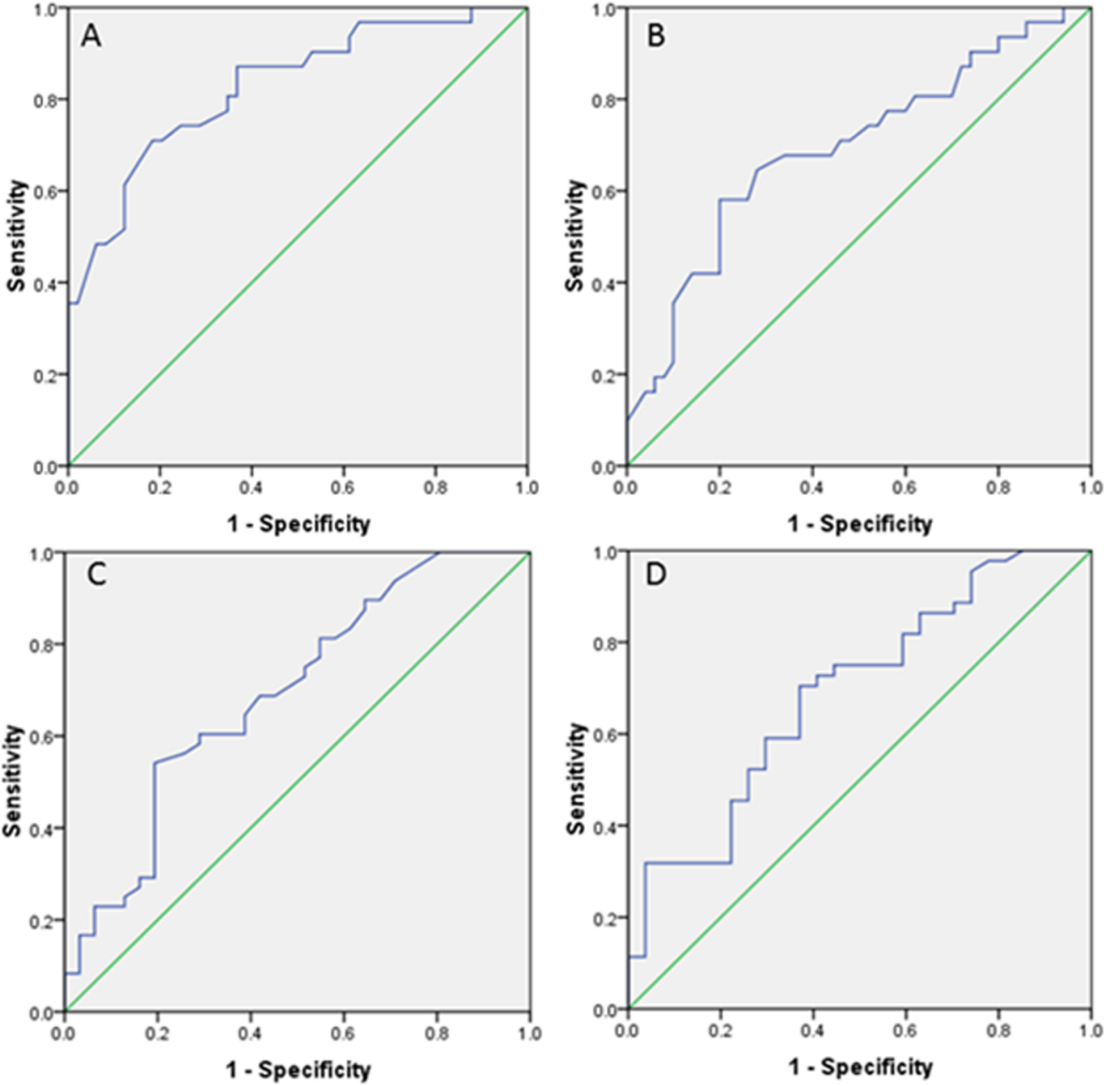
Receiver operating characteristic curve (ROC) of ALB, HGB, ESR and CRP.

**Fig 2:**
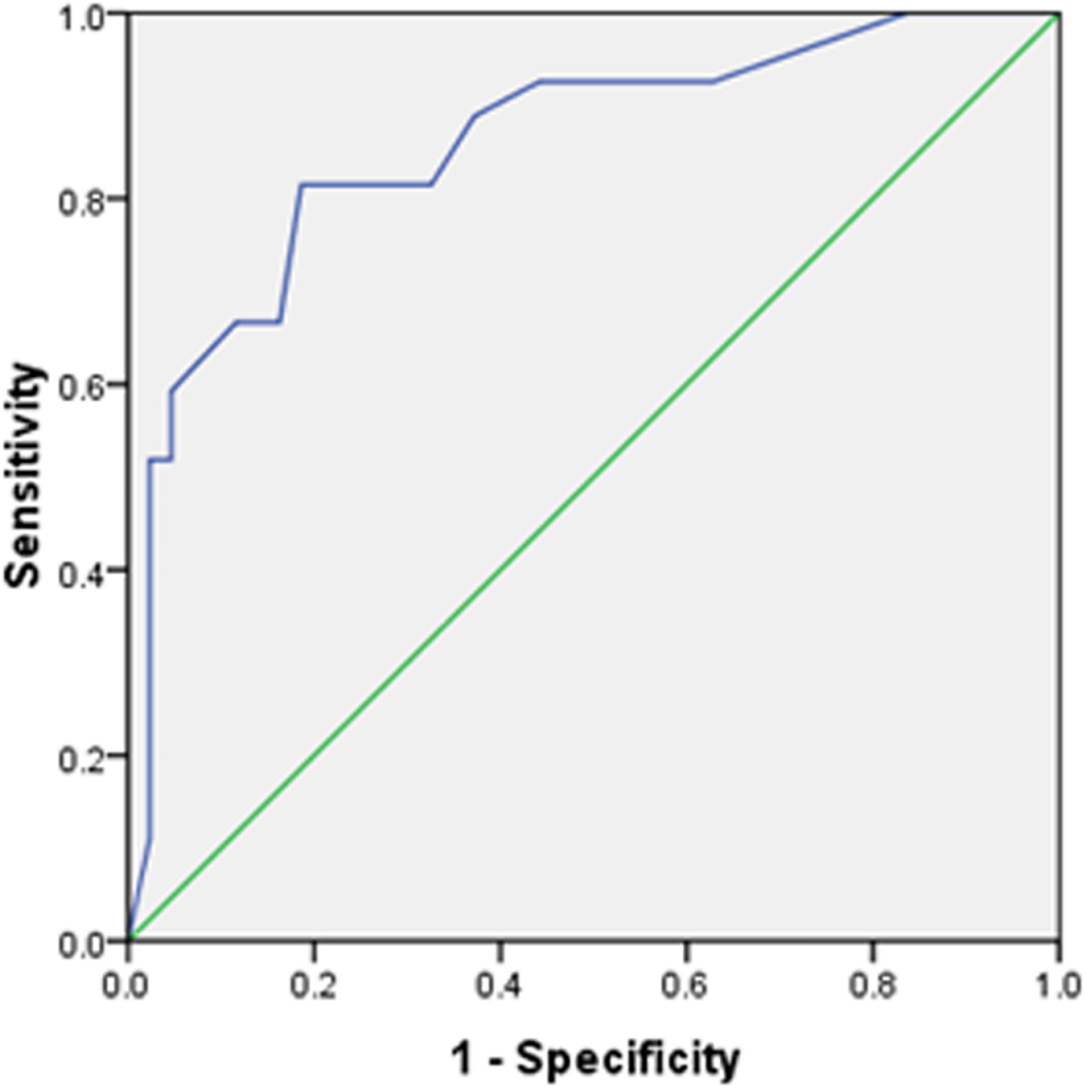
ROC of the scoring system.

Age, cough, headache, nausea, HGB, ALB, ESR, and CRP were significantly different between the two groups. Therefore, we performed multivariate logistic regression to analyze these factors. Eleven patients were removed because 10 had missing data for CRP and 1 had missing data for ESR (Fig 1). The analysis showed that only serum ALB in the equation had a significant value (OR = 1.29, 95% CI 1.09–1.52, *P* = 0.00, **Table 3**). Receiver operating characteristic curve (ROC) analysis of ALB revealed an AUC of 0.83 (95% CI 0.73–0.92, *P* = 0.000). A cut-off level at 38.85 g/L had a sensitivity of 71.0% and a specificity of 81.6% (**Fig 1A**). Other variables not in the equation could affect the model (*P* < 0.05) except age (*P* = 0.12, **Table 3**). Univariate logistic regression analysis for these seven factors also showed a significant value (*P* < 0.05) except CRP (*P* = 0.052, **Table 4**). ROC analysis of HGB revealed an AUC of 0.69 (95% CI 0.58–0.80, *P* = 0.004). A cut-off level at 131.5 g/L had a sensitivity of 58.1% and a specificity of 80.0% (**Fig 1B**). ROC analysis of ESR revealed an AUC of 0.69 (95% CI 0.57–0.81, *P* = 0.004). A cut-off level at 38 mm/H had a sensitivity of 54.2% and a specificity of 80.6% (**Fig 1C**). ROC analysis of CRP revealed an AUC of 0.69 (95% CI 0.56–0.82, *P* = 0.007). A cut-off level at 5.35 mg/L had a sensitivity of 70.5% and a specificity of 63.0% (**Fig 1D**). Because the values of the β coefficient (B value) in HGB, ALB, CRP and ESR were the result of continuous variables and were much lower than the three dichotomous variables, we converted them into dichotomous variables at their cut-off points to obtain a similar B value. After the conversion, CRP had a significant value (*P* < 0.05, **Table 4**). We established a scoring system composing of these seven factors according to their B values (**Table 5**). The score standards were as follows: B value from ±1.00 to ±1.50 counted ±1.5; from ±1.50 to ±2.0 counted ±2; and >2.0 counted 2.5. ROC analysis of the scoring system revealed an AUC of 0.86 (95% CI 0.77–0.95, *P* = 0.00). A cut-off score at 0.75 had a sensitivity of 81.5% and a specificity of 81.4%. A score above 2.75 achieved a specificity of 95.3% with a sensitivity of 59.2%, and a score below -0.75 might exclude CNS tuberculosis, with a sensitivity of 88.9% and specificity of 62.7% (**Fig 2**). The scoring system was verified and evaluated by the K-S test and the t-test. K-S test analysis showed that the scores of the two groups were normally distributed. The independent-sample t-test comparison showed a significant difference between the two groups (95% CI -5.54 –-2.80, *P* = 0.00, **Table 6**).

**Table 3:** Multivariable logistic regression of the eight factors.

Binary Variables	Variables Not in the Equation		Variables in the Equation			
	Score	*P*	B	Significance	OR	95.0% CI for OR
Age	2.42	0.12	-0.35	0.17	0.97	0.92 to 1.02
cough	6.21	0.01	-0.07	0.92	0.93	0.23 to 3.87
nausea	7.97	0.00	0.30	0.73	1.36	0.24 to 7.51
headache	13.02	0.00	0.68	0.37	1.97	0.45 to 8.57
HGB	5.54	0.02	0.02	0.23	1.02	0.99 to 1.05
ALB	21.13	0.00	0.25	0.00	1.29	1.09 to 1.52
ESR	5.48	0.02	0.01	0.70	1.01	0.98 to 1.04
CRP	4.25	0.04	-0.03	0.14	0.97	0.93 to 1.01

HGB = hemoglobin; ALB = serum albumin; ESR = erythrocyte sedimentation rate; CRP = C-reactive protein

**Table 4:** Odds ratio of the seven factors.

Binary variable	B	*P*	OR	95.0% CI for OR
cough	-1.261	0.009	0.283	0.111 to 0.725
nausea	1.738	0.004	5.684	1.759 to 18.37
headache	2.008	0.000	7.445	2.719 to 20.389
HGB	0.035	0.013	1.035	1.007 to 1.064
ALB	0.218	0.000	1.243	1.119 to 1.382
ESR	-0.025	0.010	0.976	0.958 to 0.994
CRP	-0.040	0.057	0.961	0.922 to 1.001
HGB§	1.712	0.001	5.538	2.049 to 14.973
ALB§	2.385	0.000	10.864	3.763 to 31.369
ESR§	-1.400	0.007	0.247	0.089 to 0.681
CRP§	-1.400	0.007	0.247	0.089 to 0.680

Cough, nausea and headache are dichotomous variables. ESR, ALB, CRP and HGB are continuous variables.

§ The continuous variables are converted to dichotomous variables according to their cut-off points. HGB, ALB, ESR and CRP cut-off value: 131.5 g/L, 38.85 g/L, 38 mm/H, and 5.35 mg/L, respectively.

**Table 5:** Score assignment of the scoring system.

	B Value	Negative(-)	Positive(+)	Cut-off Point
cough	-1.3	0	-1.5	
nausea	1.7	0	2	
headache	2.0	0	2	
HGB§	1.7	0	2	131.5 g/L
ALB§	2.4	0	2.5	38.85 g/L
ESR§	-1.4	0	-1.5	38 mm/H
CRP§	-1.4	0	-1.5	5.35 mg/L

Positive scores for the seven factors are based on the B value of the logistic regression.

§ B values of ESR, ALB, CRP are converted from measurement data to count data according to the cut-off point. The score standard is: B from ±1.00 to ±1.50 counts ±1.5, ±1.50 to ±2.0 counts ±2, and >2.1 counts 2.5.

**Table 6:** Independent-sample t-test for the scoring system.

Group	n	Mean±s	K-S test	*P*	Difference (95% CI)
A	43	-1.62±2.67	1.07	0.000	-5.54 to -2.80
B	27	2.56±2.98	0.59		

## Discussion

The fact that only 81 suitable cases were selected from a hospital specializing in tuberculosis over a 4-year period might be related to the lower morbidity of miliary tuberculosis (which accounts for approximately 1–2% of all TB cases) and the limitation of cases being from only one hospital [36]. The morbidity of CNS tuberculosis in all miliary tuberculosis cases was 22.5% in our study, similar to previous reports of 10% to 30% [37–42].

In our study, we found a male preponderant tendency in patients with miliary tuberculosis alone, which was contrary to a previous study [43], but a female preponderant tendency in miliary tuberculosis in patients with CNS tuberculosis, which was consistent with another study [44]. Tuberculous meningitis affects all ages and is thought to be more frequent in children with miliary tuberculosis, but it is not mentioned in miliary tuberculosis complicated with CNS tuberculosis. Our study showed that the ages of patients with miliary tuberculosis alone were distributed from several months to 80 years (data not show), with an average age of 30 years, and had a tendency to centralize from 10 to 30 years, which was consistent with previous reports [7, 31, 37, 45]. On the contrary, the youngest patient was 11 years old, and the oldest was only 49 years old in the miliary TB patients with CNS tuberculosis in our study (data not show). The average age of these patients was 24 years old and was significantly lower than in those with miliary tuberculosis alone. Patients with miliary tuberculosis alone usually suffer from fever, cough, sputum, night sweats, fatigue, and shortness of breath, which are rarely accompanied by symptoms such as emaciation, headache and chest pain [21, 22, 46, 47]. When these patients complicated with CNS tuberculosis, headache might be a normal symptom, and more clinical symptoms such as nausea and vomiting could be observed [6, 44]. Similar to other previous reports [6, 21, 22], in our study, fever was the most common symptom in both diseases, but there was no significant difference between them. Cough was more common in patients with miliary tuberculosis, whereas headache and nausea were more frequent in miliary tuberculosis patients with CNS tuberculosis. The incidences of night sweats, fatigue and emaciation (15%, 21%, 5%, respectively) in our study were not as common as in previous reports and did not show significant differences between the diseases [46].

Anemia is very common in TB patients, with a large prevalence range from 32% to 86% [48]. Miliary TB, alone or complicated with CNS tuberculosis, is also associated with anemia, is more serious than normal TB, and is often a dominant lethal factor [21, 43, 47, 49–51]. Patients with miliary TB alone in our study had a morbidity of anemia more than 50%, which was much higher than that found in those complicated with CNS tuberculosis (less than 30%), and the median level of HGB with miliary TB alone was significantly lower than that in miliary TB with CNS tuberculosis. Hypoalbuminemia and elevated levels of ALT, AST and GGT suggest a liver function injury, and hypoalbuminemia and raised ALT were considered to be independent predictors in the development and outcome of severe, active TB [21, 22, 44, 52]. Our study showed that the median levels of ALT, AST and GGT were higher than the upper limits of the normal range in both diseases, but there were no significant differences between them. Hypoalbuminemia was very common in miliary tuberculosis in our study and was consistent with previous reports [21, 22], but it was very rare in miliary tuberculosis patients with CNS tuberculosis. The median level of ALB in patients with miliary tuberculosis was significantly lower than in those complicated with CNS tuberculosis. Similar to previous reports [21, 22], neither disease affected kidney function.

ESR is a commonly used, non-specific test to evaluate acute and chronic inflammation and is markedly elevated in active TB patients; ADA is a diagnostic predictor in active TB, and CRP is also an indicator in the acute inflammatory response. These tests are elevated in TB patients, particularly in active TB patients who are HIV positive, and thus have diagnostic value [23, 25–28, 53, 54]. In our study, elevated median levels of ADA, ESR and CRP were observed in patients with miliary tuberculosis alone, but only an elevated median level of ESR was observed in those complicated with CNS tuberculosis. The median levels of ESR and CRP in miliary tuberculosis alone were significantly higher than those in patients complicated with CNS tuberculosis.

Factors including age, cough, nausea, headache, HGB, ALB, ESR and CRP have been proved to be significantly different between patients with miliary tuberculosis and miliary tuberculosis patients complicated with CNS tuberculosis. Multivariate logistic regression also showed that ALB in the equation has a positive significant value for CNS tuberculosis, and the remaining factors not in the equation had significant value for CNS tuberculosis except age. Additionally, the multivariate logistic regression and univariate logistic regression for these factors showed significant values for CNS tuberculosis except age (CRP might be considered to have a significant value for its P = 0.052 and the previous multivariate logistic regression result). Thus, we concluded that ALB is the independent risk predictor, and other factors, including cough, nausea, headache, HGB, ESR and CRP are non-independent predictors for CNS tuberculosis in patients with miliary tuberculosis.

In the ROC analysis, we found that ALB could be used to differentiate miliary tuberculosis and miliary tuberculosis with CNS tuberculosis for its high AUC of 0.83, with a sensitivity of 71.0% and specificity of 81.6% at the cut-off point of 38.85 g/L. Each factor, including HGB, ESR and CPR, could also be used to perform such an evaluation, although doing so would result in a lower AUC. Furthermore, univariate logistic regression analysis showed that cough, ESR, and CRP are protective predictors, whereas nausea, headache, ALB, and HGB are risk predictors for CNS tuberculosis in patients with miliary tuberculosis. These factors might provide a possible way to evaluate CNS tuberculosis in patients with miliary tuberculosis, similar to many scoring systems, including APACHE II, PSI, SOFA and CURB-65, which are used to evaluate the severity and prognosis of clinical patients. Hence, we tried to establish a scoring system that included cough, nausea, headache, ALB, HGB, ESR and CRP according to their B values. Because the latter four factors are measurement variables and their B values are much lower than are those of the former three dichotomous factors, we converted them to dichotomous variables at their cut-off points and performed univariate logistic regression again. We found that CRP had a significant value after the conversion and that they all had approximate absolute B values. Then, we assigned values to these factors according to their B values and established the scoring system. The scoring system is a mathematical model based on the summation of the assigned values of the seven predictors. The ROC of the scoring system showed an AUC of approximately 82% at the cut-off point of 0.75 to confirm CNS tuberculosis in patients with miliary tuberculosis, with a specificity of 81% and a sensitivity of 81%. The scoring system has a specificity of 95% to confirm the diagnosis at the score of 2.75. It also has a sensitivity of nearly 90% to exclude CNS tuberculosis in patients with miliary tuberculosis. Furthermore, the scoring system proved to be normally distributed by the K-S test, which indicated that the system could be used to evaluate CNS tuberculosis in clinical patients with miliary tuberculosis. The system also showed that miliary tuberculosis patients with scores ≤ -0.75 or ≥ 0.75 might be excluded or confirmed with the complication of CNS tuberculosis without the need for head CT/MRI scan or cerebrospinal fluid examinations, whereas those with scores in the range of -0.75 to 0.75 would need the examinations.

### Limitations

One limitation is that all patients with miliary tuberculosis alone or complicated with CNS tuberculosis whom we selected were from one hospital. One more limitation is that the scoring system is just a statistical model to evaluate the possibility of CNS tuberculosis in patients with miliary tuberculosis and could not replace the CSF examination. A further limitation is that we had a small sample, retrospective study, and the results must be supported by prospective studies with large sample sizes.

### Conclusion

Our findings demonstrate that ALB is the key independent risk predictor and that other factors including cough, nausea, headache, HGB, ESR and CRP are non-independent predictors for CNS tuberculosis in patients with miliary tuberculosis. Our newly established scoring system should be useful for evaluating whether a miliary tuberculosis patient complicates with CNS tuberculosis, which could help doctors avoid excessive investigation.

## Supporting information

S1 Dataset Original data for Tables 1 and 2. (XLSX)
