# Correction: A scoring system to effectively evaluate central nervous system tuberculosis in patients with miliary tuberculosis

**DOI:** 10.1371/journal.pone.0182713

**Published:** 2017-08-02

**Authors:** The PLOS ONE Staff

## Notice of Republication

This article was republished on June 19, 2017, to correct an error that was introduced in the title during the typesetting process. The publisher apologizes for the error.

## Supporting information

S1 File Originally published, uncorrected article. (PDF)

S2 File Republished, corrected article. (PDF)
